# The typicality effect in basic needs

**DOI:** 10.1007/s11229-022-03859-9

**Published:** 2022-09-08

**Authors:** Thomas Pölzler, Ivar R. Hannikainen

**Affiliations:** 1 Department of Philosophy, University of Graz, Attemsgasse 25/II, 8010 Graz, Austria; 2 Department of Philosophy I, University of Granada, Campus de la Cartuja, 18011 Granada, Spain

**Keywords:** Basic needs, Typicality effect, Conceptual analysis, Experimental philosophy, Prototype theory

## Abstract

According to the so-called Classical Theory, concepts are mentally represented by individually necessary and jointly sufficient application conditions. One of the principal empirical objections against this view stems from evidence that people judge some instances of a concept to be more *typical* than others. In this paper we present and discuss four empirical studies that investigate the extent to which this ‘typicality effect’ holds for the concept of *basic needs*. Through multiple operationalizations of typicality, our studies yielded evidence for a strong effect of this kind: (1) Participants tended to recall the same core examples of the concept in a free-listing task. (2) They judged some basic needs to be more typical than others. (3) The items that were judged to be more typical were listed more frequently in the free-listing task. (4) These items were listed earlier on in the free-listing task. (5) Typical basic needs, as well as non needs, were classified faster than atypical basic needs in a reaction time study. These findings suggest that the concept of basic needs may have a non-classical (e.g., exemplar or prototype) structure. If so, the quest for a simple and robust intensional analysis of the concept may be futile.

**Supplementary Information:**

The online version contains supplementary material available at 10.1007/s11229-022-03859-9.

## Introduction

According to the so-called Classical Theory, concepts are mentally represented by individually necessary and jointly sufficient application conditions (Hull, 1920; Katz & Fodor, 1963; Locke [1690] 1979). Variants of this theory have dominated scholars’ thinking about the internal structure of concepts since ancient times. In recent decades, however, the Classical Theory has been challenged. Some of the most pressing objections against it have been based on a line of psychological research that was conducted initially by Eleanor Rosch^1^ in the 1970s, documenting a so called “typicality effect” (Rosch, 1973, 1975, 1978; Rosch & Mervis, 1975).^2^

According to the Classical Theory, all things that fall under a concept ought to count equally as instances of the concept. For example, anyone who fulfills the conditions of being unmarried and male equally deserves to be called a “bachelor”. But this prediction contradicts Rosch et al.'s evidence, which suggests that with regard to some concepts people in fact judge some instances of these concepts to be more typical than others. For example, they judge that sparrows are a better example of the concept *bird* than penguins; if asked to name instances of the concept, they more often name sparrows than penguins; and they are faster in classifying sparrows as birds than penguins (Rips et al., 1973; Rosch, 1973, 1975; Rosch et al., 1976).

In light of these findings, many psychologists nowadays hold that concepts are mentally represented in non-classical ways. So called prototype theories, for example, claim that categorization is a function of similarity to a concept’s abstract average of typical instances, its so called “prototype”. People apply a concept (such as *bird*) to things that share enough of the sufficiently *weighty*—though often not necessary—features of typical instances (such as the characteristics “flies”, “nests in trees”, “sings”, etc.) (e.g., Rips et al., 1973; Hampton, 1979, 1981, 2006; Rosch, 1973, 1975, 1978; Rosch & Mervis, 1975).

Rosch and followers’ research on the typicality effect has been claimed to carry implications for philosophy. Most importantly, this research has been leveraged against the way in which philosophers have traditionally sought to analyze concepts. Suppose concepts are mentally represented in non-classical ways, e.g., as prototypes. Philosophers such as Ramsey (1992), Kornblith (2007) and Stich (1992, 1993) have argued, very roughly and with some variation, that in this case there can be no conceptual analysis that is both (1) simple (i.e., amenable to a limited number of necessary and jointly sufficient application conditions) and (2) robust (i.e., which admits of no intuitive counterexamples).

Philosophers have responded to the above challenge against traditional conceptual analysis by pointing out that the typicality effect has only been found for concepts such as *bird*, *furniture*, *weapon* or *vehicle*. In their view, this does not warrant the inference that the internal structure of concepts that are the subject matter of philosophers’ conceptual analyses show this effect as well. For example, Weatherson, one proponent of this “specificity defense”, writes:

Philosophers aren’t particularly interested in terms like ‘weapon’, so these experiments only have *philosophical* interest if the results can be shown to generalise to terms philosophers care about[, i]n other words, if [it] can be shown that terms like ‘property’, ‘justice’, ‘cause’ and particularly ‘knows’ are cluster concepts, or family resemblance terms (Weatherson, 2003, p. 19).^3^

In fact, even most philosophers who have raised the above challenge against traditional conceptual analysis have conceded that the typicality effect has not been established for (their targeted) philosophical concepts. They have therefore presented their arguments as only hypothetical: *if* findings of typicality generalize to concepts such as *knowledge*, *responsibility*, and *consciousness then* analyses of these concepts cannot be both simple and robust (Ramsey, 1992; Stich, 1992, 1993).^4^ So is the specificity defense indeed successful?

It is important to note that contrary to Weatherson, Ramsey, Stich and others, there already exists some evidence against the specificity defense. Psychologists have investigated typicality effects in a number of abstract concepts, at least some of which may be deemed philosophical or philosophically relevant; e.g., the concepts *lie* (Coleman & Kay, 1981), *work of art*, *belief*, *crime*, *just decision*, *property*, *instinct*, *rule*, *science* and *kind of work* (Hampton, 1981); *extraversion* and *introversion* (Cantor & Mischel, 1979); *schizophrenia*, *affective disorder* and other psychiatric diagnostic concepts (Cantor et al., 1980); and *female* and *male* (Arcuri, 2001).^5^ For most of these concepts (though not all)^6^ the effect was sufficiently pronounced for researchers to infer a non-classical conceptual structure.

At the same time, it is true that we still lack a clear indication of whether the typicality effect holds for many other philosophical concepts. Hence, further research on typicality judgements about philosophical concepts is clearly called for. This paper will present novel research of this kind. As the object of our case study, we focus on a concept that arises in a wide range of philosophical contexts, in particular in normative ethical discussions about issues such as domestic justice (e.g., Copp, 1995; Miller, 1999), global justice (e.g., Brock, 2009), intergenerational justice (e.g., Meyer & Pölzler, 2022; Wolf, 2009), climate justice (e.g., Gough, 2017), international development (e.g., Braybrooke, 1987; Doyal & Gough, 1991), and sustainability (e.g., WCED, 1987)—namely the concept of *basic needs*.

The absence of research on the typicality effect in this context is striking. Philosophers in this area have attempted to provide simple and robust analyses in terms of individually necessary and jointly sufficient application conditions (e.g., Copp, 1995; Doyal & Gough, 1991; Braybrooke, 1987; Brock, 2009; Miller, 1999; for an overview see Pölzler, 2021). Yet, if we introspect about the concept of basic needs, a few concrete instantiations of the concept readily come to mind (e.g., water, food, shelter), and it seems plausible that these core basic needs serve as the basis for an abstract prototype. The hypothesis that we attempt to test here thus is that just as the concepts of a bird, a lie, a work of art, science, etc., the concept of basic needs exhibits effects of typicality.

Our paper reports support for this hypothesis throughout four pre-registered empirical studies.^7^ Participants often recalled the same examples of basic needs when asked to freely list instances of the concept (Study 1). They robustly judged some instances to be more typical than others (Studies 2a and 2b). The instances that were recalled more frequently and earlier in the free-listing task were also rated as more typical than instances that were listed less frequently or later. Finally, participants were faster in classifying typical basic needs, as well as non-needs, than atypical ones (Study 3).

In what follows we will present and discuss our studies in detail. Then we will argue that our findings suggest that the concept of basic needs has a non-classical (e.g., prototypical) structure, and that this provides us with reason to rethink philosophers’ traditional approach to analyzing this concept. We will close by briefly discussing our findings’ broader implications for the specificity defense and for philosophical methodology.

## Study 1: Free-listing task

Our first step in testing the hypothesis of a typicality effect in basic needs employed a “free-listing task”. Free-listing tasks are an established qualitative method (Quinlan, 2017; Weller & Romney, 1988) that has been commonly used in typicality effect research (e.g., Hampton, 1981; Rosch, 1975; Wang et al., 2016). These tasks ask participants to report what they freely associate with a given concept.

The results of free-listing tasks have mainly been analyzed in terms of how *frequently* certain items are associated with the concept under investigation (How many participants listed item *i*?). In addition, the *rank order* of each item is relevant as well (On average, which position does item *i* take in participants’ list, i.e., is it listed first, second, third, etc.?); and researchers have also developed indices that combine frequency and rank to yield a composite index of how cognitively salient certain aspects of a concept are.

Free-listing tasks mainly contribute to testing typicality effect hypotheses in combination with other studies (see Studies 2a, 2b and 3). But in the context of our research these tasks are also independently helpful. They reveal the language that laypeople use in addressing certain features or instances of a concept, which can help formulate subsequent tasks in ways that are familiar to participants (within certain constraints of efficiency and theoretical adequacy). Moreover, free-listing tasks might also provide some initial indication of how a concept is cognitively structured. If a concept is represented in terms of exemplars or prototypes, then participants should mostly freely associate examples with it (rather than conditions for the application of the concept).

### Participants

For our study we recruited 102 participants via Prolific Academic, an online crowdsourcing platform. As inclusion criteria, we required that participants: (1) reside in the United States or Great Britain, (2) speak English as their first language, (3) have completed anywhere between 10 and 200 studies^8^ with (4) an approval rate no lower than 90%. 7 participants were excluded from analysis because they finished the survey in less than 02:00 min.^9^

Of the remaining 95 participants, ages ranged from 19 to 72 (Mean = 30.1, SD = 11.5), and 39% were women. 68% of participants identified as Caucasian, 8% as Asian, 8% as Hispanic, and 1% as African-American. Median yearly income ranged between $10,000 and $19,999 and participants were slightly left-leaning (mean political orientation on nine-point-scales from 1 = “extremely liberal” to 9 = “extremely conservative”: Social = 3.63; Economic = 4.07) and generally non-religious (47% reported ‘None’ as the strength of religious beliefs on a four-point scale).

### Methods

Free-listing tasks typically only involve a brief and simple question. They ask participants to list things or features that they associate with the concept under investigation, in order to document participants’ free associations without introducing bias (Quinlan, 2017). Hence, participants of our study were asked the following question about basic needs:

What are **basic needs**? What comes to mind when you think about this concept? In the space provided below, please list at least ten things that you associate with basic needs. Separate each element in the list using a semicolon (‘;’).

The study also involved another small task, administered after the above one, that was part of a related but different research project and therefore will not be reported here. Finally, participants were asked a series of demographic questions.

### Results

When asked what comes to mind when they think about basic needs, all participants (95 out of 95) provided examples of the concept, such as “water”, “food” or “shelter”. No participant associated any application conditions with the concept of basic needs, such as “necessary to prevent harm” or “necessary to be autonomous”.

Supplementary Table 1 displays the frequency and mean rank of each item that was listed at least thrice. Examination of these results suggested that participants often referred to the same basic needs using close synonyms (e.g., “mobility” and “transportation”), descriptions involving varying levels of detail (e.g., “water” and “drinkable water”), plural and singular forms (e.g., “social contact” and “social contacts”), etc. We therefore undertook a qualitative coding exercise (see coding criteria in Online Appendix) with the goal of subsuming different formulations of the same basic need. An intercoder reliability check revealed an overall Cohen’s κ of 0.96.

Table 1 provides summary statistics resulting from our coding exercise: the frequency of each item (i.e., by how many participants the item was listed), the mean position of each item (i.e., whether on average, the item was listed first, second, third, etc.), and the cognitive salience of each item, which we calculated employing the following formula devised by Sutrop (2001): frequency/(sample size * mean position).

**Table 1:** Frequency, mean position and cognitive salience (frequency/(sample size * mean position)) of all items that were mentioned by at least three participants in Study 1, ordered by frequency

Item	Frequency	Mean position	Cognitive salience
Food	95	1.82	1
Water	84	2.20	0.731
Shelter	69	3.04	0.435
Health	32	4.88	0.126
Clothes	26	4.42	0.113
Companionship	22	4.77	0.088
Hygiene	21	3.95	0.102
Love	20	4.50	0.085
Sleep	19	4.16	0.088
Air	17	3.29	0.099
Money	16	5.13	0.060
Safety	15	4.00	0.072
Education	14	4.79	0.056
Warmth	12	3.75	0.061
Family	9	5.00	0.035
Happiness	7	4.57	0.029
Freedom	7	5.14	0.026
Friends	6	6.67	0.017
Electricity	5	4.60	0.021
Mobility	5	5.40	0.018
Survival	4	1.25	0.061
Employment	4	5.00	0.015
Entertainment	4	5.00	0.015
Goals	4	6.25	0.012
Phone	3	4.00	0.014
Religion	3	4.33	0.013
Rest	3	4.33	0.013
Comfort	3	5.33	0.011
Culture	3	5.67	0.010
Respect	3	8.00	0.007

Frequency and mean position were negatively correlated, Spearman’s ρ = − 0.47, *p* = 0.009, i.e., the more participants listed an item the earlier on it appeared on their lists.

### Discussion

Our first study asked participants what they freely associate with the concept of basic needs. In line with the hypothesis that the concept has a non-classical internal structure, participants exclusively associated examples with it. In fact, there was not one single participant who provided a description of any potential abstract application condition of *basic needs*.

Our data also provides some first indication that some instances of *basic needs* are considered to be more typical than others. In terms of frequency as well as rank and cognitive salience, food, water and shelter led the field by a comfortable margin. A second group of items (health, clothes, companionship, hygiene, love, sleep, air, money, safety, education, and warmth) were also mentioned relatively often and relatively early on, but significantly less so than the aforementioned. Finally, some participants also listed additional putative instances of *basic needs* that seem to be less typical.

As said, in addition to providing some preliminary evidence about typicality and the structure of *basic needs*, Study 1 is mostly relevant in conjunction with other research. We will move on to this research now.

## Study 2a: Typicality ratings (convenience sample)

For a concept to show the typicality effect means that some of its instances are more typical than others. The most obvious and common way to test this hypothesis is by asking people to judge various instances of the concept in terms of their typicality. For example, in some previous studies people were asked how typical they thought robins, sparrows, eagles, penguins, etc. are for the concept of birds; or how typical they thought cars, boats, horses and skis are for the concept of vehicles (Rips et al., 1973; Rosch, 1975; Rosch & Mervis, 1975).

If the typicality effect holds for a concept, then we should find that people robustly judge certain instances of this concept (e.g., sparrows, cars) to be more typical than other instances (e.g., penguins, skis) (e.g., Rips et al., 1973; Rosch, 1973). Moreover, items that are judged to be more typical should be listed more frequently and should rank higher in corresponding free-listing tasks (e.g., Rosch, 1975; Rosch et al., 1976). To test these predictions regarding the concept of basic needs, we conducted a second study that asked participants to rate the typicality of several potential basic needs.

### Participants

Based on the same prescreening criteria as in Study 1, we recruited 315 participants via Prolific Academic. 13 participants were excluded from analysis because they completed our survey in less than 02:00 min or failed more than one of three attention/comprehension checks. These checks required them to (1) move the slider for a particular item to 3 (tolerance: ± 0.2), (2) move the slider for a particular item to 6 (tolerance: ± 0.2), and (3) rate “champagne” as a poor example of the concept of basic needs (rating higher than 5.5 on a seven-point scale from 1 = “very good example” to 7 = “very poor example”).

Of the remaining 302 participants, ages ranged from 18 to 73 (Mean = 27.1, SD = 8.71), and 79% were women. 78% of participants identified as Caucasian, 8% as Asian, 5% as African-American, and 4% as Hispanic. Median yearly income ranged between $20,000 and $29,999, and participants tended toward a liberal-leaning (mean political orientation on nine-point scales from 1 = “extremely liberal” to 9 = “extremely conservative”: Social = 3.09; Economic = 3.87) and non-religious (38% reported ‘None’ as the strength of their religious belief on a four-point scale) worldview.

### Methods

Participants were asked to rate 30 items (potential basic needs) in terms of their typicality for the category of basic needs. The items were derived as follows: First, we included all 21 items that were listed at least five times in Study 1, according to our coding exercise (see Table 1). Second, to increase variance in typicality and the philosophical relevance of our research, we included additional nine items that both (a) were listed by at least one participant in Study 1, and (b) have been mentioned as an example of basic needs in the philosophical literature. The complete list of 30 items can be found in Table 2.

**Table 2:** Mean typicality ratings in Studies 2a and 2b on a seven-point scale from 1 = “very good example” to 7 = “very poor example”, ordered by ratings in 2a

Item	*Typicality*		*Factor loadings*		
	Study 2a	Study 2b	F1	F2	F3
Water	1.27	1.35	0.96		
Food	1.31	1.43	0.94		
Air	1.35	1.39	0.91		
Shelter	1.42	1.60	0.86		
Sleep	1.44	1.54	0.88		
Health	1.57	1.66	0.77		
Safety	1.70	1.81	0.67	0.41	
Survival	1.75	1.66	0.72		
Rest	1.90	2.04	0.57		
Warmth	2.13	2.03	0.50	0.43	
Hygiene	2.21	2.21	0.43	0.59	
Clothing	2.21	2.25	0.42	0.47	
Happiness	2.27	2.62			0.53
Freedom	2.34	2.14	0.43	0.50	
Love	2.36	2.81			0.70
Education	2.52	2.52		0.58	
Family	2.63	2.88			0.58
Companionship	2.64	2.88			0.72
Justice	2.75	2.58		0.62	
Mobility	2.76	2.63		0.51	
Money	2.84	3.03		0.52	
Friends	2.98	3.25			0.74
Respect	3.00	3.17		0.49	0.50
Electricity	3.04	3.04		0.67	
Nature	3.10	3.04			0.45
Employment	3.16	3.23		0.62	
Sex	3.92	3.91			0.54
Entertainment	4.17	4.43			0.49
Art	4.71	5.05			0.41
Religion	5.04	5.40			

Results on the factor structure are based on combined data from Studies 2a and 2b. Factor loadings below 0.40 are omitted

As said, for each of these items participants were asked to rate them in terms of how typical they are for the category of basic needs. The particular formulation of our instructions was adapted from Rosch (1975) as follows:

It is generally thought that humans have **basic needs**. However, it is not clear what things are good examples of these needs. For instance, is music a typical example of a basic need? In what follows you will be presented with a number of items. Please rate **how good an example** these items are of the category of basic needs. “1” means that you feel the item is a very good example of your idea of what basic needs are. “7” means that you feel the item fits very poorly with your idea of what basic needs are (or is not a member of this category at all). Use the other numbers on the scale to indicate intermediate judgments. We are not interested in why you believe that something is a basic need or whether others would agree with you. **Just give us your personal opinion**.

After this task participants were asked a number of demographic questions.

### Results

As predicted by the typicality effect hypothesis, some of the presented items (e.g., water, food, air, shelter and sleep) were judged to be significantly more typical of basic needs than others (e.g., employment, sex, entertainment, art and religion). This variation in typicality across items was confirmed in a repeated-measures ANOVA, *F*_(32, 9614)_ = 318.51, *p* < 0.001. Table 2 displays mean typicality ratings for each item.

**Fig. 1:**
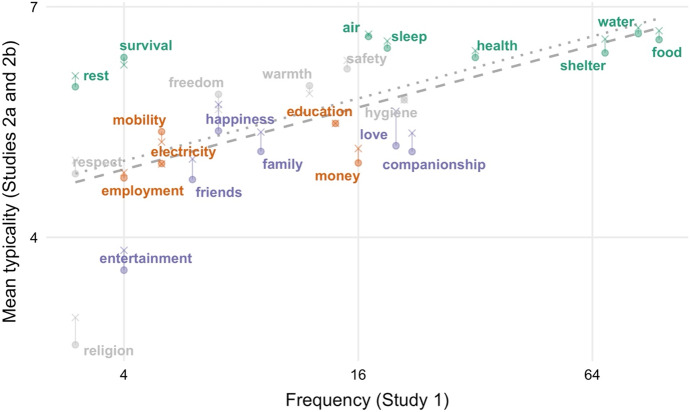
Mean typicality ratings in Studies 2a and 2b against frequency in Study 1. Item colors represent main factor loadings, with Factor 1/Red being interpreted as physiological needs, Factor 2/Green being interpreted as psychological or personal development needs, and Factor 3/Blue being interpreted as social or affective needs. Typicality (on the y-axis) is reverse-coded, such that higher values indicate greater typicality

**Fig. 2:**
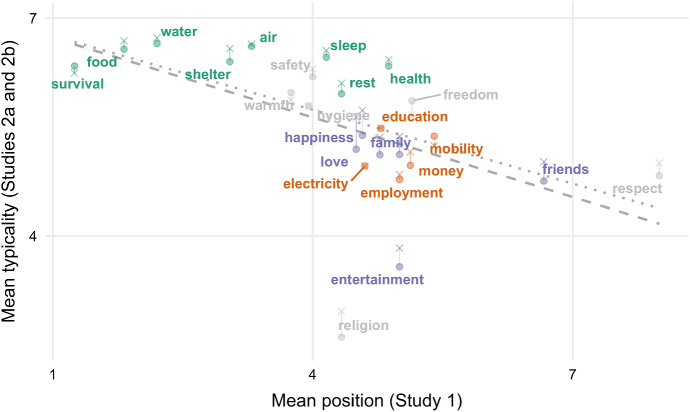
Mean typicality ratings in Studies 2a and 2b against mean position in Study 1. Item colors represent main factor loadings, with Factor 1/Red being interpreted as physiological needs, Factor 2/Green being interpreted as psychological or personal development needs, and Factor 3/Blue being interpreted as social or affective needs. Typicality (on the y-axis) is reverse-coded, such that higher values indicate greater typicality

We also examined the predictions that more typical basic needs would be listed (a) more frequently and (b) before less typical basic needs in Study 1. Both of these predictions were confirmed. More typical basic needs were included more often than less typical ones, Spearman’s ρ = 0.67, and earlier on in participants’ lists, Spearman’s ρ = − 0.73 (both *p*s < 0.001). Only a few items were judged to be somewhat more typical (e.g., water, food, air, shelter) or less typical (e.g., entertainment, religion) than their cognitive salience in Study 1 would have predicted. Figures 1 and 2 below provide a visualization of this relationship between mean typicality rating (on the vertical axes) and frequency and mean position (on the horizontal axes).

### Discussion

The hypothesis that there is a typicality effect in *basic needs* generates three predictions with regard to typicality ratings: (1) people should judge some instances of basic needs to be significantly more typical than others, (2) instances that are judged to be more typical should be listed more frequently in corresponding free-listing tasks, and (3) instances that are judged to be more typical should be listed earlier on in corresponding free-listing tasks. Study 2a (in combination with Study 1) provided evidence for all three of these predictions.

Just as in the free-listing task, a group of physiological needs (water, food, air, shelter, etc.) received the highest typicality ratings. They were thought to be the best examples of basic needs. Survival, air, sleep and some other of these physiological needs were regarded as even more typical than their cognitive salience would have predicted. This might partly be explained by the following two facts: (1) the fulfillment of these needs happens more or less automatically or as a by-product of fulfilling other needs, and (2) this fulfillment does not allow for as much variation. For example, people think less about how to breathe or how to sleep than about what to eat. This means that air and sleep are less cognitively salient (as measured by Study 1), even though judged to be more typical (as measured by Study 2).

Conversely, the needs for entertainment and religion were judged to be *less* typical than their frequency and mean position in the free-listing task would have predicted. This too may be partly explained by the effort expended in satisfying the needs and the diversity in means by which these needs are met. There are various ways of being entertained (ranging from partying to reading a book) and spiritually fulfilled (e.g., meditating or attending a church service). These needs might thus be more salient to people than corresponding typicality judgements would give reason to expect.

## Study 2b: Typicality ratings (nationally representative sample)

Obtaining typicality ratings is the most consequential part of our research on the typicality effect in *basic needs*. Not only do these ratings provide independent evidence about our hypothesis; results about them also combine with both Study 1 and Study 3 to test further important predictions. For this reason we decided to re-run Study 2a with a nationally representative sample. Comparing convenience and nationally representative samples also enabled us to evaluate the correlation between typicality ratings across different sampling methods.

### Participants

We recruited 392 participants via Prolific Academic. They were prescreened in the same way as in Study 1 and 2a, with the exception that all participants were residents of the United Kingdom. The sample of our study was representative of the United Kingdom adult population in terms of sex, age and ethnicity. Fourteen participants were excluded from analysis because they completed the survey in less than 02:00 min or failed more than one of the three attention/comprehension checks (which were the same as in Study 2a).

Of the remaining 378 participants, ages ranged between 18 and 76 (Mean = 45.6, SD = 15.4), and 51% were women. 80% of participants identified as Caucasian, 9% as Asian, 5% as Black, and 4% as Mixed. Median yearly income ranged between £20,000 and £29,999, and participants tended slightly toward a liberal-leaning (mean political orientation on nine-point scales from 1 = “extremely liberal” to 9 = “extremely conservative”: Social = 3.84; Fiscal = 4.37) and non-religious (51% reported ‘None’ as the strength of their religious belief on a four-point scale) worldview.

### Methods

The methods of this study were identical to those of Study 2a (see Sect. 3.2).

### Results

As in Study 2a, participants’ ratings of the typicality of the 30 items that they were presented with varied substantially according to a repeated-measures ANOVA, *F*_(32, 12064)_ = 405.10, *p* < 0.001 (see Table 2).^10^ We also replicated the correlation between typicality ratings on the one hand and frequency and mean position on the other. In particular, the better of an example for basic needs an item was judged to be, the more frequently it was listed in the free-listing task, Spearman’s ρ = 0.62, *p* = 0.001; and the earlier on it was on average listed in this task, Spearman’s ρ = − 0.72, *p* < 0.001 (see Figs. 1, 2).

Moreover, Study 2b allowed us to examine the extent to which by-item typicality ratings correlated across different samples; in particular, across the convenience (2a) and nationally representative (2b) samples. It turned out that the ratings were almost perfectly correlated, Pearson’s *r* = 0.99, *p* < 0.001 (see also Table 2).

Finally, to examine the factor structure of our list of basic needs, we carried out a maximum-likelihood factor analysis with varimax rotation on the combined data of Studies 2a and 2b (*n* = 673). Three factors exhibited eigenvalues above one (7.92, 4.63, and 4.18, with the 4th factor dropping to 0.52), and explained a total of 56% of variance in typicality ratings (0.26, 0.15, and 0.14, with the 4^th^ factor contributing an additional 0.02). Table 2 reports the factor loadings of each item in the list.

### Discussion

Study 2b successfully replicated the results of Study 2a drawing on a nationally representative sample, thus increasing these results’ generalizability. The three predictions generated by the hypothesis that there is a typicality effect in *basic needs* were again all supported: (1) participants judged some items (such as water, food, air and shelter) to be more typical of basic needs than others (such as entertainment, art and religion), (2) items that were judged to be more typical were listed more frequently in the free-listing task, and (3) items that were judged to be more typical were listed earlier on in the free-listing task.

In our discussion of Study 2a, we pointed out that some physiological needs (e.g., water, air, sleep, and survival) were rated as better examples of basic needs than their cognitive salience in Study 1 would have predicted; and others (e.g., entertainment and religion) were rated as worse examples—presumably, because fulfilling these needs does/does not happen rather automatically and admits of high variation in the means of fulfillment. In Study 2b this tendency was even stronger. Most notably, air and sleep, which ranked only at seventh and ninth place in terms of their cognitive salience in the free-listing task, were now judged to be the second and third best example of basic needs, respectively.

Our factor analysis (based on data from Studies 2a and 2b) provided evidence concerning the structure of people’s intuitions regarding basic needs. Three factors were identified and accounted for more than half of the variance in typicality ratings. Considering the items that loaded selectively onto a single factor (see Table 2) encouraged an interpretation in terms of three families of basic needs—which is also common in the philosophical literature (e.g., Miller, 1999; Reader & Brock, 2004).

At a broad level, Factor 1 (e.g., water, food, and sleep) appeared to capture *physiological* basic needs: things that are required for survival and normal bodily functioning. Factors 2 and 3, in contrast, referred to non-physiological kinds of basic needs. The items that loaded onto Factor 2 (e.g., education, employment and mobility) may be described as *psychological* needs, or needs that relate to personal development. The items that loaded onto Factor 3 (e.g., love, companionship and friends) were tied to *social* or *affective* needs.

Finally, it may be helpful to say a few words about how our investigation of the typicality of basic needs relate to what is arguably the most well-known psychological theory on this matter, namely Maslow’s “hierarchy of needs” (1943, 1954).

There are important differences between Maslow’s research objectives and methods and ours. Maslow did not investigate how *good an example* of basic needs certain items are judged to be. Rather, he was interested in the ordering of different kinds of needs and how they *motivate* human beings. In particular, he claimed that this motivation unfolds in distinct stages, first attending to unmet needs for physiological and safety needs, and only then moving on to belongingness and love, esteem, and self-actualization. But that a certain kind of need is accorded priority in terms of its satisfaction does not necessarily mean that it would be perceived as typical of basic needs. For example, sex—which Maslow ranks as a highly motivating physiological need—received low typicality rankings in our studies (the fourth-lowest of all items, see Table 2).

There are also important methodological differences. Maslow (1954) arrived at his ordering of needs by examining the biographies of a small number of people who he considered to be highly “self-actualized”: mostly white Western males such as Einstein, Lincoln, Jefferson and Beethoven. Leaving aside the apparent shortcomings of this method, our typicality effect hypothesis is not limited to such a particular population, but applies to all those who have the concept of basic needs—which is why the studies in this paper were conducted on samples of ordinary people.^11^

## Study 3: Response times

A last prediction of the typicality effect hypothesis concerns response times. If the typicality effect holds for a concept, then people should be faster in classifying (a) typical basic needs and (b) non-basic needs than they are in classifying atypical basic needs (instances that are judged to be neither good nor poor examples of the concept). For example, examining the concept *bird*, Rosch (1975) found that classifying a sparrow as a bird (typical instance) takes significantly less time than classifying a penguin as a bird (atypical instance). This has been treated as evidence for a typicality effect in the concept *bird*, and consequently for this concept’s non-classical internal structure (Rosch, 1975).^12^

Our third study tested whether the above response time prediction holds for the concept of basic needs. That is, we ask whether people are faster in classifying (a) typical instances of basic needs (e.g., food, water, air) and (b) non-instances of the concept (e.g., champagne, jewelry) than they are in classifying atypical instances (e.g., entertainment, sex, employment).^13^

### Participants

Employing the same prescreening criteria as in Studies 1 and 2a, we recruited 313 participants via Prolific Academic. Nine participants were excluded from analysis because they categorized five or more items within 100 ms (which we deemed insufficient time to read and classify an item) or because they categorized two or more of the following items as basic needs: “champagne”, “jewelry”, “enemies”. In the remaining sample of 304 participants, ages ranged between 18 and 65 (Mean = 30.1, SD = 10.4), and 73% were women. We did not request additional sociodemographic information as part of this study.

### Methods

Our study was developed and conducted with the online survey tool Gorilla Experiment Builder (www.gorilla.sc). While online recordings of response times generally do not achieve quite as much precision as lab-based solutions, Gorilla was found to be among the two best performers in terms of these recordings, with variability of reaction time measurements on almost all browsers and operating systems < 5 ms (Bridges et al., 2020). The main advantage of using this web-based solution was that participants did not need to go to any lab or to download any software on their computers.

Participants were presented with the same 30 potential basic needs that were used for Studies 2a and 2b (see 2.2) as well as with the 3 aforementioned items that were used for control purposes (“champagne”, “jewelry”, and “enemies”). At the beginning of the study, participants received the following information:

This task is about the category of basic needs. You will be presented with a number of items (single words). For each of these items, please classify them as quickly as possible as either a basic need or not a basic need. The task will take about 2 minutes.

The following page provided more detailed instructions:

Put your index fingers on the ‘f’ and the ‘j’ keys of your keyboard. The items will appear one-by-one in the middle of the screen. When you think that the item is a basic need (‘yes’) then press the ‘f’ key. When you think that the item is not a basic need (‘no’) then press the ‘j’ key. Once you press the space bar the task will start immediately. So please be prepared. Try to go as fast as you can.

After this set of instructions, participants were shown the aforementioned 30 + 3 items in a randomized order. Each target item was displayed individually on its own slide, and participants categorized the items as either basic needs or not basic needs by pressing the corresponding buttons.

### Results

Our main analysis concerns the relationship between typicality ratings in Studies 2a and 2b and response times. Some items in Studies 2a and 2b were rated as highly typical of basic needs (e.g., food, water, air) and some were rated as atypical (e.g., entertainment, sex, employment). However, participants did not judge any of the items to be clear *non-instances* of the concept of basic needs. On a seven-point scale from 1 = “very good example” to 7 = “very poor example” only entertainment (4.17), art (4.71) and religion (5.04) exceeded the midpoint.

**Fig. 3:**
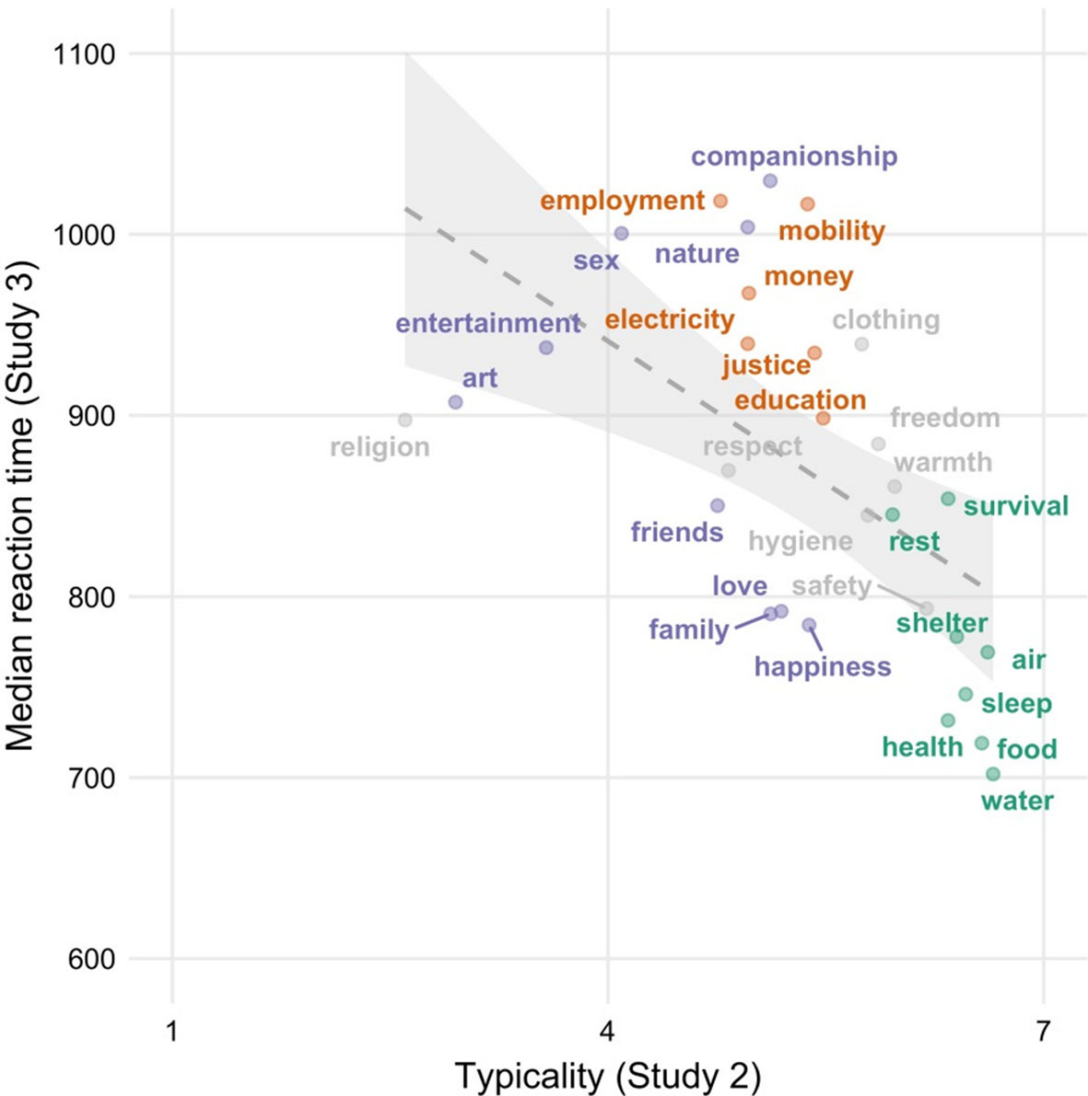
Typicality (in Study 2) against median response time (in Study 3). Typicality (on the x-axis) is reverse-coded, such that higher values indicate greater typicality

This absence of non-instances means that our response time prediction is a negative correlation between typicality (in Studies 2a and 2b) and latency (in Study 3). Indeed, we found that participants were faster in classifying typical instances of basic needs than they were in classifying atypical instances, Spearman’s ρ = − 0.69, *p* < 0.001 (see Fig. 3).

The above analysis does not reveal whether people are faster in classifying non-basic needs than they are in classifying atypical basic needs. With the inclusion of control items, Study 3 provided the opportunity to test this further prediction. All of our control items were classified as not being basic needs in Study 3: “champagne” = 96% “no” responses, “jewelry” = 97% “no” responses, and “enemies” = 96% “no” responses.^14^ Since classifications in Study 3 were otherwise almost perfectly correlated with typicality ratings in Study 2, Pearson’s *r* = 0.97, *p* < 0.001, the relationship between these classifications (including our control items) and response times should hence not be linear but inverse U-shaped, with shorter response times when mean classification judgments approached 0 (“no” = not a basic need) or 1 (“yes” = a basic need), and longer response times when they elicited disagreement (i.e., approached 0.5).

**Fig. 4:**
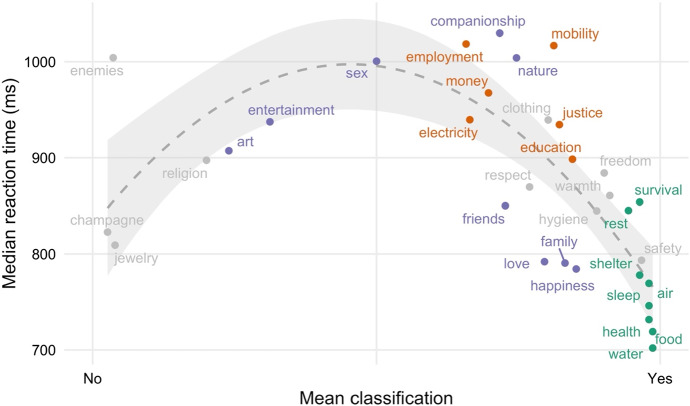
By-item mean categorization against median response time in Study 3

This prediction was also borne out: A quadratic model of classification judgments (AIC = 6737) provided better fit to the data than a simple log-linear model (AIC = 6826), χ^2^_(df = 1)_ = 91.29, *p* < 0.001. In this model, we observed a negative effect of reaction time, *OR* = 0.31, *z* = − 10.45, *p* < 0.001, such that ‘Yes’ responses were faster than ‘No’ responses. Critically, this model also uncovered a positive quadratic effect, *OR* = 1.13, *z* = 6.59, *p* < 0.001, indicating that participants were slow to issue classification judgments for those items that elicited disagreement. Figure 4 provides a visualization of the inverse U-shaped relationship between classifications and response times.

Finally, the prediction that people are faster in classifying typical instances of basic needs than items that they judge to be atypical received additional support when looking at the cognitive salience indicators in Study 1. Participants who freely associated a particular item with the concept of basic needs, and in particular who listed this item early on, presumably took this item to be a (very) good example of the concept. Basic needs that were recalled (a) more *frequently* and (b) *earlier* in our free-listing task should thus be classified faster under time pressure.

**Fig. 5:**
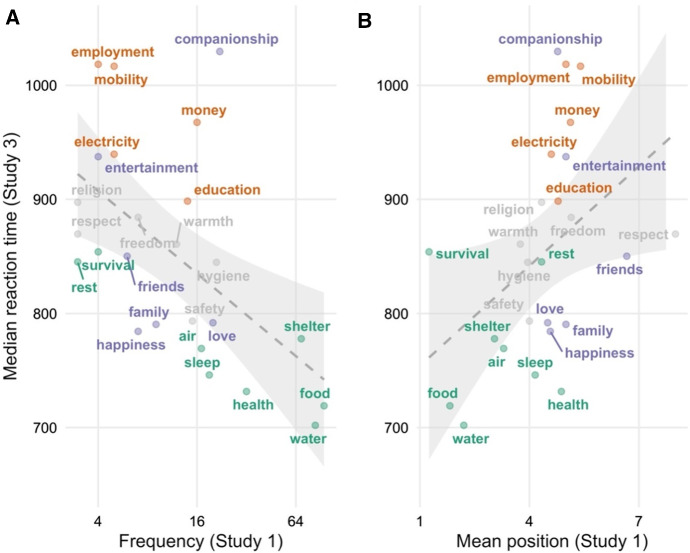
Median reaction time (in Study 3) against frequency (A) and mean position (B) (in Study 1)

Indeed, we found that response times in Study 3 correlated negatively with frequency, Spearman’s ρ = − 0.57, *p* = 0.003, and positively with mean position, Spearman’s ρ = 0.56, *p* = 0.003, as illustrated in Fig. 5a and b.

### Discussion

Study 3 tested the prediction that people are faster in classifying (a) typical basic needs and (b) non-basic needs than they are in classifying atypical items. Our results lend support to this prediction.

Examining the relationships between typicality judgements (in Studies 2a and 2b) and response times (in Study 3) showed that those items perceived as the best examples of basic needs (e.g., food, health, water, air) were classified faster than more atypical items (e.g., sex, entertainment, electricity or employment). This result is also supported by the finding that both cognitive salience indicators in Study 1—which might be thought of as ‘behavioral’ measures of typicality—predicted faster reaction times in Study 3. Finally, items that were judged not to be basic needs at all required less processing time than items that yielded disagreement in the categorization task (i.e., atypical basic needs).

Deviations from the above trends are potentially explained by our choice of items and labels. For example, “enemies”, though almost unequivocally regarded as not a basic need, shows a median response time of > 1000 ms, close to that of divisive items such as “sex” or “employment” (see Fig. 4). This unusually elevated latency may reflect the fact that it is rather uncommon, strange or even amusing to consider whether humans might have a basic need for enemies—at least, one might pause for a moment to think about this proposition.

Response times for “companionship”, “mobility”, “employment” and some other items may have been affected by our wording, which is consistent with some participants’ word choice in the free-listing task, but inconsistent with the preferred labels of others. For instance, the notion of “companionship” was referred to in a variety of different ways, such as “social contacts”, “relationships”, or “someone to be with” (see Sect. 10.2). Perhaps participants would have been faster in processing this item if it had been described in their preferred terms. Finally, the lower general familiarity and greater length or complexity of some labels may have inflated response times for the respective items as well.

## General discussion

Some philosophers have argued that psychological studies on the typicality effect have important implications for how to analyze concepts (e.g., Kornblith, 2007; Ramsey, 1992; Stich, 1992, 1993). One way in which such arguments have been criticized is by claiming that these studies have only investigated non-philosophical concepts, and that we cannot generalize from these concepts to philosophical ones (e.g., Weatherson, 2003). Previous research (e.g., Coleman & Kay, 1981; Cantor & Mischell, 1979) has already cast some initial doubt on this “specificity defense” to which the research in this paper is meant to add.

In our studies, we directly investigated the extent to which the typicality effect holds for an important philosophical concept in normative ethics, namely the concept of basic needs (which, intuitively, seems to be a particularly likely candidate for showing such an effect). Our studies provided several strands of evidence for a strong typicality effect in *basic needs*. (1) Participants tended to recall the same core examples of the concept in a free-listing task. (2) They judged some basic needs to be more typical than others. (3) The items that were judged to be more typical were listed more frequently in the free-listing task. (4) These items were listed earlier on in the free-listing task. (5) Typical basic needs, as well as non needs, were classified faster than atypical basic needs in a reaction time study.

These findings are limited in several respects. Most importantly, we only surveyed native English speakers residing either in the United States or Great Britain. Only in one study (Study 2b) our sample was nationally representative in terms of sex, age and ethnicity; and other potentially relevant demographic characteristics (such as income or education) were non-representative across all studies. The generalizability of our findings to other populations, especially to non-English speakers and members of other cultural groups, hence remains unclear (see Henrich et al., 2010).

Bearing this limitation in mind, we think that our findings nevertheless hold important lessons both for (1) the psychology of the concept of basic needs and (2) the philosophical methodology to be employed in analyzing this concept.

### Psychological implications

The main psychological implication of our findings concerns the internal structure of the concept of basic needs. As explained in the introduction, psychologists widely assume that the Classical Theory fails to explain typicality effects. On this theory each instance of a concept represents the concept equally well (as it satisfies the conditions for falling under the concept equally well), and should thus be regarded as equally typical. Our evidence of varying typicality in potential instances of *basic needs* accordingly suggests that this concept might not be structured in a classical way, i.e., it might not be constituted by a belief about the concept’s individually necessary and jointly sufficient application conditions.

There are several alternative psychological theories of concepts that purport to explain typicality effects better than the Classical Theory, among them (1) the exemplar theory (Brooks, 1978; Medin & Schaffer, 1978), (2) the theory theory (e.g., Carey, 1985; Gopnik & Meltzoff, 1997) and (3) the prototype theory (Hampton, 2006; Rosch, 1973, 1975, 1978; Rosch & Mervis, 1975). Our results do not privilege any of these theories in any clear way. In what follows we will proceed under the assumption that all things considered, some version of the prototype theory captures the internal structure of *basic needs* best. This is because it is the alternative theory that has so far received most attention among philosophers (not at least because of its anticipation in Wittgenstein, see fn. 2), and that leaves most initial hope for traditional conceptual analysis to be salvageable after all (which seems less likely on the assumptions of exemplar theory or theory theory).

Prototype theories state that a person who attempts to decide whether an item falls under a concept compares this item to a stored prototype that was formed by abstracting certain characteristics, as well as the relative statistical prominence of these characteristics, from the most typical instantiations of the concept. For example, a person will classify penguins as falling under the concept *bird* if they judge that penguins share enough sufficiently weighty prototypical characteristics of birds, such as “flying”, “singing”, “nesting in trees”, or “laying eggs”. If, on the other hand, the person judges that penguins do not resemble the prototype closely enough then they will refrain from categorizing them as birds.

Importantly, none of the statistically prevalent characteristics that form a prototype need to have the status of a necessary condition, as they are postulated by the Classical Theory (see Laurence & Margolis, 1999). People categorize items as falling under concepts even though these items sometimes lack some or many of these concepts’ prototypical characteristics. The instances of a concept (e.g., of the concept *bird*) consequentially do not all share the *same* characteristics (they do not all fly and sing and nest in trees, see penguins); rather, as Wittgenstein remarked with regard to the concept of games, we see a “complicated network of similarities overlapping and criss-crossing” (PI §66).

What are the characteristics that constitute the prototype of *basic needs*? And what is their relative weight? Our studies do not provide evidence about these questions. One might speculate—and we have in fact begun to conduct follow-up research on this question^15^—that the prototype of the concept of basic needs includes characteristics along the following lines: (a) “being necessary for survival”, (b) “being necessary to avoid serious harm”, (c) “being necessary for flourishing”, etc.

In any case, the prototype structure of *basic needs* offers a promising explanation of the typicality-related findings in our research. Variation in whether candidate items constitute typical examples of a basic need (Studies 2a and 2b), for example, can be explained by the supposition that these items manifest varying numbers of central characteristics of the prototype. “Food” and “water”, for example, plausibly share more of these characteristics than “love”. Assuming the above hypothetical list of characteristics, they share both (a), (b) and (c), while “love” (on standard interpretations) only shares (c).

The finding that typical basic needs and non-basic needs were classified faster than atypical basic needs (Study 3) is well explained by *basic needs*’ hypothesized prototype structure too. If the concept is structured in this way, then computing whether an item falls under the concept involves going through its prototypical characteristics until one concludes that the item shares them to a sufficient extent (or doesn’t). As typical items like “food” and “water” share many of the prototype’s characteristics, the threshold of categorization sufficiency is reached fast (e.g., after checking only (a) or perhaps (a) and (b) above). To determine whether an atypical item like “love” is a basic need, in contrast, more characteristics need to be examined (e.g., not only (a) and (b), which are not shared by “love”, but also (c)), which takes longer.

### Philosophical implications

To some it may seem doubtful that a psychological theory of a concept’s internal structure can have implications for how to philosophically analyze this concept. After all, in conducting such analyses philosophers are not concerned with how we *think* about the concept but with what it *means*. That said, Ramsey (1992), Stich (1992, 1993) and Kornblith (2007) have argued that traditional philosophical analysis involves certain presuppositions about concepts’ internal mental structure—presuppositions that are hard to reconcile with the Classical Theory. Here we will take up and elaborate on this argument with regard to the concept of basic needs.

Like with any other concept, philosophers have so far attempted to analyze *basic needs* in terms of simple sets of individually necessary and jointly sufficient application conditions. For example, a number of ethicists and political philosophers have argued that *x* is a basic need if and only if having/being/realizing *x* is required to avoid serious harm such as in the sense of impairments of one’s autonomy (e.g., Copp, 1995; Doyal & Gough, 1991), rational agency (e.g., Brock, 2005; Copp, 1996) or ability to function socially (e.g., Braybrooke, 1987; Miller, 1999).^16^

These analyses have, among others, been justified and criticized on the basis of philosophers’ intuitions about cases. For example, Hassoun (2016) has criticized the above standard definition by arguing that intuitively, people sometimes need things that are not required for them to avoid serious harm:

Intuitively, some of the things people need they need not merely to avoid harm but in order to flourish. […] In some developing countries there are ten year old children who are working and will not receive a secondary school education. […] these children will not be harmed by failing to receive this education: They are not made worse off than they were before if they are not educated. But, intuitively, at least most of these children do need education. (Hassoun, 2016)^17^

Hassoun’s argument here is that even in cases in which a thing is not required to avoid serious harm we sometimes have the intuition that the thing is needed; therefore, the avoidance of serious harm cannot be a necessary condition for (*basic*) *needs* (as entailed by the standard analysis).

However, justifying and criticizing analyses of *basic needs* on the basis of conceptual intuitions only seems to make sense if these intuitions align relatively closely with some simple set of necessary and sufficient conditions. On a prototype theory of the concept this likely will not be the case. Our intuitions about what qualifies as an instance of *basic needs*—including the intuitions of philosophers—are influenced by comparisons of items with the concept’s prototype; and items can be sufficiently similar to this prototype in virtue of having not just the same one or two or three individually necessary characteristics but in virtue of having a far broader range of non-necessary characteristics. To illustrate, item 1 may intuitively strike us as a basic need because it has characteristics (a) and (b), item 2 because it has characteristics (a), (d) and (e), item 3 because it has characteristics (b), (c) and (f), and so on.

This means that on a prototype account it is likely that we will be able to come up with intuitive counterexamples to *any* analysis according to which any characteristic is necessary for *basic needs*—not only the analysis targeted by Hassoun, i.e., in terms of the avoidance of serious harm, but also her own analysis of basic needs as anything that enables a person to live a minimally good human life (2016); analyses according to which x is a basic need if and only if x required for survival and rational agency (Schuppert, 2013), analysis according to which x is a basic need if and only if x is required for understanding and exercising our rights and liberties (Wolf, 2007); and so on. We just have to identify some item that, even though it does not share the particular characteristic that is claimed to be necessary, shares a sufficient number of the other characteristics that constitute the *basic needs* prototype.^18^

One way of dealing with this challenge to traditional analyses of the concept of basic needs would be to allow for these analyses to become more complex. If an analysis provided a full or near-full list of the concept’s prototypical characteristics, including their particular weights and a categorization threshold, this would restore its immunity to counterexamples. For example, very roughly, one could determine category membership on the basis of a list including (a) “being necessary for survival” (0.8), (b) “being necessary to avoid serious harm” (0.7), (c) “being necessary for flourishing” (0.2), and so on; as well as a specific threshold value (1.0). The obvious downside of this response is that it makes identifying basic needs and integrating the analysis into normative ethical theories a much more difficult and messy endeavor.

A perhaps more attractive solution is to stick to simple analyses but no longer purport that these analyses capture *basic needs* in full and are hence immune to all counterexamples. It might be argued that this is what has been happening all along. When philosophers have claimed that, e.g., x is a basic need if and only if x is required to avoid serious harm this is to be understood as a partial or imperfect analysis that has been regarded as useful in a particular context. We are not sure about this interpretation. Certainly, conceptual analysis is not generally understood in such a way; and the above example by Hassoun shows that it is often thought that analyses of *basic needs* in particular do purport to be immune to all counterexamples. In any case, even if our argument did not necessitate changes in the self-understanding of proponents of traditional analyses of *basic needs*, it would at least be a call for being clearer and more explicit about the limitations of these analyses.

There are several ways in which our conclusions might be resisted. Among others, critics might claim that (1) some version of the Classical Theory can explain the typicality effect reasonably well after all (Smith & Medin, 1981), (2) even though this theory does not provide a good explanation of the typicality effect, its explanatory advantages with regard to other relevant phenomena still render it superior to non-classical theories (see Pinker & Prince, 1988; Jackendoff, 1983); (3) some other non-classical theory, e.g., exemplar theory or theory theory, is better supported than prototype theory, and is more compatible with the way in which *basic needs* has traditionally been analyzed; (4) striving for simple and robust analyses in the sense of an ideal is pragmatically useful, as it leads to better understandings of aspects of a concepts (Sandin, 2006); (5) philosophical analyses do not purport to analyze concepts at all but philosophical phenomena directly (Deutsch, 2021), (6) philosophers and psychologists talk about different things when they talk about concepts (Machery, 2009), (7) philosophical analyses are not (or not primarily) about describing the content of these concepts but rather about prescribing it (Cappelen, 2012).

Here we cannot defend our argument against these objections. Our conclusions are hence tentative. We would only like to suggest that discussants of *basic needs* should consider the possibility that this concept is mentally represented in a non-classical way, and does not admit of traditional analyses in terms of sets of individually necessary and jointly sufficient application conditions that are both simple and robust.

In fact*,* we believe that all philosophers who conduct traditional analyses would be well-advised to consider whether their target concepts exhibit a non-classical structure, and if yes, what this implies for their methodology. In light of the typicality effect results of this paper as well as of psychological studies on the structure of concepts such as *lie*, *work of art*, *female*, and *male*, and in light of other kinds of evidence for non-classical structures of *free will* (Berniūnas et al., 2021; May, 2014) and moral concepts (Park, 2013), the specificity defense has started to crumble. It no longer seems justifiable to assume, without argument, that all or most philosophical concepts are constituted by beliefs in individually necessary and jointly sufficient application conditions.

Hopefully, our case study will inspire more empirical and theoretical research on this important matter, so as to ensure the adequacy of the method of conceptual analysis for different philosophical concepts.

## Supplementary Information

Below is the link to the electronic supplementary material.

Supplementary file1 (DOCX 27 kb)

## Footnotes

1 According to Rosch (1975), her research on typicality (and prototype semantics more generally) was inspired by Wittgenstein’s idea of “family resemblance”, as it figures most prominently in §66–68 of his *Philosophical Investigations*.

2 In an important review of the field, Laurence & Margolis call this line of research the “most influential argument” against the Classical Theory (1999, p. 24). Another helpful review can be found in Machery (2009).

3 The original quotation by Weatherson involves two errors that have been corrected here, namely a full stop after “about” and the missing “it”.

4 For example, Ramsey notes: “the thesis I’ll defend is a conditional one concerning what follows if certain empirical accounts of concept representation are correct” (Ramsey, 1992, p. 59) Similar qualifications can also be found in Stich: “Although there has been an enormous amount of work on concepts and categorization in recent years, there has been no systematic empirical study of intentional categories—categories like believing that p, or desiring that q. Thus at present we can only speculate about what such an investigation would reveal.” (Stich, 1992, p. 149) “To the best of my knowledge, there have been no empirical studies aimed at exploring the mental representation of moral concepts like justice or responsibility. Nor has anyone looked carefully at the cognitive structures underlying our ability to use categories like things it is morally acceptable to kill for food.” (Stich, 1993, p. 224).

5 In addition to the above-mentioned direct investigations, some researchers have also proposed indirect empirical arguments for the non-classical structure of certain philosophical concepts (see Park, 2013 for moral concepts) or have conducted empirical studies about this structure that do not test for typicality (see Berniūnas, 2021; May, 2014 for the concept of free will).

6 No sufficiently strong typicality effect was found for *belief*, *just decision*, *property*, *instinct*, and *rule* (Hampton, 1981).

7 The studies’ preregistrations are accessible via the following anonymized view-only links: Study 1: https://osf.io/yjwdq/?view_only=3a95107b92484bf39ba179f89fde8df9. Study 2a: https://osf.io/cr9fj/?view_only=a620ee091bf4485ba6213a6095c5162c. Study 2b: We did not generate a preregistration for 2b, as the experimental design was identical to that of 2a. Study 3: https://osf.io/96ujy/?view_only=4d9e99b89d3448d8a400a92347a94d41. All studies were approved by the IRB of the first author’s university.

8 This criterion was included to make sure that participants were familiar with online surveys but did not complete them professionally or semi-professionally.

9 The exclusion criteria of this study as well as of all other studies reported in this paper (2a, 2b and 3) were stated in the respective preregistrations, see fn. 7.

10 Variation in typicality ratings may be due to *item* variance (i.e., different items being rated more or less typical) and/or *participant* variance (i.e., different participants providing higher or lower typicality ratings). The results of the repeated-measures ANOVAs indicate that variation in typicality ratings varies significantly across items. The effect of item accounted for 43% of variance (pseudo-*R*^2^) in both Studies 2a and 2b, and the inclusion of the participant variable improved model fit to 58% and 59% (in Studies 2a and 2b, respectively). Consistent with the typicality effect hypothesis, a comparison of the *fixed* (item variance) versus *total* (item-plus-participant variance) pseudo-*R*^2^ values revealed that typicality ratings primarily reflect variation within individuals (i.e., across items), and not across individuals.

11 Besides Maslow, many other researchers have studied (basic) needs from a psychological perspective as well, e.g., in the contexts of well-being and development (e.g., Constanza et al., 2007; McGregor et al., 2009) or self-determination theory (Ryan & Deci, 2000). In our understanding, none of these studies—illuminating as they are in other respects—provide significant evidence about the specific question of whether the concept of basic needs shows a typicality effect either.

12 In her studies Rosch did not include highly atypical items. She only looked at the contrast between “typical” items (which we here call “highly typical”) and “atypical” ones (which we here call “ambivalent”).

13 The typicality effect hypothesis also entails that people make more classification errors under time pressure when it comes to ambivalent compared to highly typical and highly atypical items, e.g., they more often classify penguins than sparrows as non-birds (e.g., Rosch, 1973; Smith et al., 1974). However, testing this prediction requires some objective standard of whether each candidate item is a category member or not (such the taxonomic classification of each species for the bird category)—which does not exist for the category *basic needs*.

14 Note that a very small number of participants (9 out of 313) were excluded because they rated more than one of the above items to be basic needs (see Sect. 5.1).

15 In this research we will attempt to specify the characteristics (including their weight) that constitute the *basic needs* prototype.

16 For a brief overview of analyses of *basic needs* see Pölzler (2021).

17 Hassoun is aware and explicitly states that this argument only works if one assumes a particular conception of harm, namely a conception according to which a person is only harmed by an action if the action makes her worse off than before; and that this conception likely will be rejected by proponents of harm-avoidance analyses of basic needs. Note also that Hassoun discusses these analyses *qua* analyses of needs, not *basic* needs in particular.

18 Further difficulties for traditional analyses of *basic needs* are introduced by the facts that prototypes vary across persons, change across individuals’ lifetimes, and involve characteristics whose weight can significantly depend on context (see Ramsey, 1992 for discussion).
