# Supplementary material for: The typicality effect in basic needs
Source: Synthese. 2022 Sep 8;200(5):382. doi: 10.1007/s11229-022-03859-9 (PMC9458586; doi:10.1007/s11229-022-03859-9)
Supplement: Supplementary file 1 — Supplementary file1 (DOCX 27 kb) [file 11229_2022_3859_MOESM1_ESM.docx]

# Appendices

## Supplementary Table 1: Free-Listing Task

| **Item** | **Frequency** | **Mean position** | **Cognitive salience** |
| --- | --- | --- | --- |
| food | 76 | 1.84 | 1.00 |
| water | 66 | 2.18 | 0.73 |
| shelter | 33 | 3.00 | 0.27 |
| love | 20 | 4.50 | 0.11 |
| clothes | 15 | 4.60 | 0.08 |
| money | 12 | 4.67 | 0.06 |
| health | 12 | 4.92 | 0.06 |
| air | 11 | 3.18 | 0.08 |
| sleep | 11 | 4.45 | 0.06 |
| education | 11 | 4.64 | 0.06 |
| healthcare | 9 | 5.67 | 0.04 |
| safety | 8 | 3.38 | 0.06 |
| warmth | 8 | 3.50 | 0.06 |
| clothing | 8 | 4.25 | 0.05 |
| house | 7 | 2.86 | 0.06 |
| family | 7 | 4.86 | 0.03 |
| home | 6 | 4.17 | 0.03 |
| happiness | 6 | 4.83 | 0.03 |
| freedom | 6 | 5.50 | 0.03 |
| friends | 6 | 6.67 | 0.02 |
| eating | 5 | 1.40 | 0.09 |
| hygiene | 5 | 3.00 | 0.04 |
| electricity | 5 | 4.60 | 0.03 |
| housing | 4 | 1.25 | 0.08 |
| drinking | 4 | 2.25 | 0.04 |
| companionship | 4 | 4.50 | 0.02 |
| entertainment | 4 | 5.00 | 0.02 |
| to eat | 3 | 1.00 | 0.07 |
| security | 3 | 3.00 | 0.02 |
| drink | 3 | 3.33 | 0.02 |
| oxygen | 3 | 3.67 | 0.02 |
| bed | 3 | 4.00 | 0.02 |
| phone | 3 | 4.00 | 0.02 |
| toilet | 3 | 4.33 | 0.02 |
| comfort | 3 | 5.33 | 0.01 |
| culture | 3 | 5.67 | 0.01 |
| sanitation | 3 | 5.67 | 0.01 |
| respect | 3 | 8.33 | 0.01 |
|  |  |  |  |

**Table 1:** Frequency, mean position and cognitive salience (frequency/(sample size * mean position)) of all items that were mentioned by at least three participants in Study 1, ordered by frequency, raw data

## Coding Criteria: Free-Listing Task

List of phrases that were merged in the process of data cleaning (see Table 1), with the first italicized item being used as the label for each set of merged items:

- *air*, breathe, breathing, oxygen, to breathe
- *clothes*, clothing, have clothes
- *companionship*, social connection, social contacts, social interaction, closeness, secure attachments, human contact, people (i.e. friends/family), human interaction, relationships, being social, social life, social support, socialization, company, someone to be with, connection
- *education*, literacy, numeracy, good education, understanding, knowledge
- *employment*, work, jobs, job security
- *exercise*, physical activities
- *family*, family support, a supporting family
- *food*, eat, eating, proteins, basic food sources to provide minimum daily rate of protein,fats and carbs and basic vitamins, bread, healthy food, to eat, sustenance
- *freedom*, autonomy,
- *friends*, friendship
- *goals*, purpose, something to do, the opportunity to chase a purpose
- *health*, free health care, health care, health insurance, health public service, health/medicine, free elderly care, good health, access to health care, a good health care system, medical supplies
- *hygiene*, sanitation, sanitary equipments, toilet, relieve yourself (by that i mean going to the wc), somewhere to clean and use a toilet, being clean, products to clean, bathroom, disinfectants, pee, paper toilet
- *mobility*, transport, transportation, car, public transportation system
- *money*, enough money to live decently, finance/currency, having a decent income, living wage
- *privacy*, private space
- *religion*, spirituality, belief
- *rest*, mental rest
- *safety*, protection, to feel safe, feeling safe, security, clean and safe environment
- *sex*, sexuality
- *shelter*, a home, roof, house, housing, to have a house or a place to stay, a house, place to rest, place to sleep, a place to live, having a place to sleep, secure place to call home, somewhere to live, have a roof over your head, home, house
- *sleep*, sleeping, bed, to sleep
- *warmth*, heat, source of heat
- *water*, drink, drinkable water, drinking, water to clean or brush, water to drink, to have water, clean water, clear and safe water, to drink water

## Individual Difference Analyses

This section reports individual difference analyses of our studies. That is, we investigated to what extent our results varied with the demographic data that we collected.

First, we conducted three Poisson regression analyses assessing whether there were individual differences in the frequency with which participants in Study 1 recalled the basic needs loading selectively onto one of three factors (see Section 4.4). None of our individual difference measures predicted the frequency of physiological (*p*s > .12), psychological (*p*s > .14), or social (*p*s > .42) basic needs.

|  | **Physiological**  χ²(6) = 4.03, *p* = .67 | | | **Psychological**  χ²(6) = 8.65, *p* = .19 | | | **Social**  χ²(6) = 3.66, *p* = .72 | | |
| --- | --- | --- | --- | --- | --- | --- | --- | --- | --- |
|  | *B* | *z* | *p* | *B* | *z* | *p* | *B* | *z* | *p* |
| Intercept | 1.41 | 5.97 | < .001 | -0.84 | -1.46 | .14 | -0.90 | -1.96 | .05 |
| Age | -0.01 | -1.56 | .12 | 0.01 | 0.84 | .40 | 0.00 | 0.15 | .88 |
| Gender* | -0.09 | -0.74 | .46 | -0.04 | -0.11 | .91 | 0.02 | 0.08 | .93 |
| Income | 0.05 | 1.46 | .14 | 0.10 | 1.24 | .22 | 0.03 | 0.37 | .71 |
| Religiosity | 0.02 | 0.26 | .80 | -0.30 | -1.47 | .14 | -0.01 | -0.08 | .94 |
| Social | -0.02 | -0.48 | .63 | -0.06 | -0.50 | .62 | 0.07 | 0.81 | .42 |
| Fiscal | 0.02 | 0.57 | .57 | -0.01 | -0.11 | .92 | 0.06 | 0.75 | .45 |

Note. *: Dummy-coding scheme with 0s as women, and 1s as men.

Next, we evaluated whether there were individual differences in typicality ratings (based on the aggregate data from Studies 2a and 2b). We observed several significant effects: of age, gender, income bracket, and religiosity.

To confirm the pattern of results in our separate multiple regressions, we conducted a single mixed-effects model, allowing every individual difference measure to moderate the effect of domain. In this model, age interacted with factor, *F*_(2, 1299)_ = 5.42, *p* = .005, revealing that biological and psychological needs were seen as more typical among older participants, but no corresponding simple effect of age was observed for social needs. Income also interacted with factor, *F*_(2, 1230)_ = 3.67, *p* = .026, such that social and psychological needs were seen as less typical among high-income participants, but no corresponding effect was observed for physiological needs. We also observed weak main effects of religiosity, *F*_(1, 651)_ = 4.77, *p* = .029, and gender, *F*_(2, 651)_ = 2.59, *p* = .076—which reflect higher typicality ratings among religious and female participants overall.

|  | **Physiological**  *F*_(7,653)_ = 2.18, *p* = .034 | | | **Psychological**  *F*_(7,649)_ = 2.96, *p* = .005 | | | **Social**  *F*_(7,648)_ = 2.00, *p* = .052 | | |
| --- | --- | --- | --- | --- | --- | --- | --- | --- | --- |
|  | *B* | *t* | *p* | *B* | *t* | *p* | *B* | *t* | *p* |
| Intercept | 6.33 | - | < .001 | 3.94 | - | < .001 | 3.38 | - | < .001 |
| Age | 0.01 | 2.75 | .006 | 0.01 | 2.47 | .014 | -0.00 | 0.33 | .74 |
| Gender | - | - | - | - | - | - | - | - | - |
| Man | -0.21 | -2.15 | .032 | -0.19 | 1.93 | .054 | -0.08 | 0.78 | .44 |
| Non-binary | 0.24 | 0.41 | .69 | 0.48 | 0.82 | .41 | 0.68 | 1.12 | .26 |
| Income | 0.00 | 0.08 | .93 | -0.04 | -2.51 | .012 | -0.04 | -2.09 | .037 |
| Religiosity | 0.06 | 1.23 | .22 | 0.09 | 1.85 | .065 | 0.11 | 2.16 | .031 |
| Social | -0.00 | -0.13 | .90 | 0.03 | 0.72 | .47 | -0.01 | -0.28 | .78 |
| Fiscal | -0.05 | -1.36 | .18 | 0.03 | 0.88 | .38 | 0.03 | 0.89 | .38 |

In separate logistic regression models, we found no effects of age or gender on classifications (in Study 3), all *p*s > .13. When modeling responses simultaneously in a single mixed-effects regression, we obtained evidence of a negative main effect of age across domains, *OR* = 0.97, *z* = -3.10, *p* = .002.

|  | **Physiological**  χ²(2) = 0.04, *p* = .98 | | | **Psychological**  χ²(2) = 1.80, *p* = .41 | | | **Social**  χ²(2) = 2.33, *p* = .31 | | |
| --- | --- | --- | --- | --- | --- | --- | --- | --- | --- |
| Classification | *B* | *z* | *p* | *B* | *z* | *p* | *B* | *z* | *p* |
| Intercept | 3.70 | - | .001 | 1.62 | - | < .001 | 1.08 | - | .004 |
| Age | -0.01 | -0.14 | .89 | -0.02 | -1.34 | .18 | -0.02 | -1.53 | .13 |
| Gender* | 0.13 | 0.15 | .88 | -0.04 | -0.12 | .91 | 0.02 | 0.09 | .93 |
|  | *F*_(2,299)_ = 4.14, *p* = .017 | | | *F*_(2,299)_ = 4.15, *p* = .017 | | | *F*_(2,299)_ = 2.15, *p* = .12 | | |
| Response Time | *B* | *t* | *p* | *B* | *t* | *p* | *B* | *t* | *p* |
| Intercept | 762.0 | - | < .001 | 946.7 | - | < .001 | 1127.5 | - | < .001 |
| Age | 3.25 | 2.52 | .012 | 7.64 | 2.85 | .005 | 2.35 | 1.04 | .30 |
| Gender* | 37.00 | 1.22 | .22 | 15.07 | 0.24 | .81 | 90.97 | 1.72 | .086 |

Note. *: Dummy-coding scheme with 0s as women, and 1s as men.
